# The Experiences of Patients With Rare Diseases in Pennsylvania: A Community-Based Study

**DOI:** 10.1177/00333549251362711

**Published:** 2025-09-07

**Authors:** Jonathan H. Sussman, Mert Marcel Dagli, Shira L. Wald, Saad S. Akhtar, Keanu Natan, Jessica A. Xu, Alexander Li, Brian Dawson, William C. Welch

**Affiliations:** 1 Department of Neurosurgery, Perelman School of Medicine, University of Pennsylvania, Philadelphia, PA, USA; 2 University of Pennsylvania School of Dental Medicine, Philadelphia, PA, USA; 3 School of Public Health, University of Pittsburgh, Pittsburgh, PA, USA; 4 Pennsylvania Rare Disease Advisory Council, Hummelstown, PA, USA

**Keywords:** rare diseases, crowdsourcing, public health, patient advocacy, health disparities

## Abstract

**Objective:**

Rare diseases collectively affect approximately 30 million people in the United States. Despite advances in genomic medicine, early diagnosis is challenging because of limited awareness of, accessibility to, and disparities in health care resources. We assessed the real-world experiences of patients with rare diseases in Pennsylvania and evaluated the effect of delayed diagnosis on psychosocial and financial burdens.

**Methods:**

The Pennsylvania Rare Disease Advisory Council conducted a Rare Disease Needs Assessment Survey from September 2020 through January 2023. The survey, distributed through multiple channels, collected responses from patients, caregivers, and rare disease advocates in Pennsylvania. We analyzed quantitative and qualitative data on diagnosis, health care access, financial burden, and psychosocial support.

**Results:**

A total of 1214 respondents participated, representing a diverse spectrum of rare diseases and demographic groups. More than half (57.8%) of respondents indicated diagnostic delays of ≥1 year, which were associated with additional misdiagnoses, increased annual spending, out-of-state travel, and reduced work and school hours; however, diagnostic delays were not associated with disease category. Many respondents (48.5%) reported >$5000 in annual spending related to care for their rare disease, and 24.9% were unable to access medications because of financial reasons. Diagnostic delays were associated with worse perspectives on the efficacy of care across multiple domains even after a correct diagnosis was achieved. Patients aged 0 to 20 years had a faster time to diagnosis than patients aged >20 years did.

**Conclusion:**

Patients with rare diseases in Pennsylvania face substantial barriers to diagnosis, specialized care, and financial support. Despite policy initiatives, gaps remain in genetic testing access, specialist availability, and psychosocial resources. Addressing these issues through improved diagnostics, expanded access to care, and targeted policy changes is essential to enhancing patient outcomes and quality of life.

Rare diseases affect a small number of individuals (defined as <200 000 people in the United States), although in aggregate they account for more than 7000 distinct conditions that affect about 30 million people in the United States and 300 million people worldwide.^1,2^ Approximately 80% of rare diseases have a heritable genetic component, and more than 90% of rare diseases do not have current US Food and Drug Administration–approved treatments.^2-4^ Early identification can be challenging because signs and symptoms may not be readily recognized by patients and health care providers, and precise diagnosis often requires expensive genomic techniques such as whole-genome sequencing.^5^ This lack of recognition among health care providers can be attributed, in part, to limited exposure to rare diseases in medical training and the vast heterogeneity of clinical presentations. These disease presentations include unusual signs and symptoms that are not elicited during typical health care visits as well as unique constellations of seemingly benign symptoms, which can lead to misattribution to more common conditions.^6,7^

Access to genomic technology and specialists who can guide patients to optimal treatment is limited in low-income and rural communities.^8^ Moreover, these disparities are compounded by the unequal distribution of health care services and medical knowledge of genetic testing in racial and ethnic minority groups.^9^ African American and Hispanic people are less likely than the general population to be offered genetic testing and may face barriers to accessing testing services.^10,11^ These barriers include limited referral from primary care providers, lower health literacy levels related to genetic testing, cost and health insurance coverage issues, language barriers, and distrust of the medical system.^12-14^ Moreover, African American and Hispanic people are less likely than people of European ancestry to be represented in genetic databases, leading to less definitive documentation about the clinical effect of genetic variants that are enriched in those populations.^15^

The collective result of these challenges is a prolonged diagnostic timeline, which has been reported to average from 4 to 8 years.^2^ The long journey needed to obtain a diagnosis for a rare disease is often termed the “diagnostic odyssey.”^16-18^ Unlike common illnesses with well-defined symptoms and tests, rare diseases can be challenging to pinpoint. The first step typically involves consulting a primary care physician or specialist. However, because the initial symptoms might be vague or mimic other conditions, patients can go through a cycle of misdiagnoses and unnecessary tests.^18^ Many rare diseases do not even have distinct diagnostic codes—240 new rare diseases were identified between 2021 and 2024, yet only 18 new *International Classification of Diseases, Tenth Revision, Clinical Modification* codes appeared in the Orphanet nomenclature files^19^—and nonspecific codes such as “other specified congenital malformation syndromes” can be linked to as many as 531 unique rare diseases.^20^ One disease entity may represent distinct underlying etiologies, while other groups of seemingly disparate disease entities may represent multiple manifestations of the same pathophysiological process, creating challenges for disease classification and nomenclature when the biological mechanisms are not well understood.^21^ Patients with rare diseases face myriad emotional and psychosocial challenges, including feelings of isolation, alienation, and uncertainty.^22-24^ The rarity of their conditions often yields limited understanding and support from their medical providers and personal communities, exacerbating feelings of frustration and distrust.^23,25^ Repeated misdiagnoses and the lack of definitive treatments create a profound emotional toll for patients, their families, and their caregivers.^22-24^

In the United States, 2 major laws benefit people with rare diseases: (1) the Orphan Drug Act (1983) incentivizes pharmaceutical companies to develop drugs for rare conditions by offering tax credits and market exclusivity^26,27^ and (2) the Rare Disease Act (2002) established the Office of Rare Diseases within the National Institutes of Health to facilitate research initiatives and disseminate information.^28^ The Commonwealth of Pennsylvania established the PA Rare Disease Advisory Council (PARDAC) in 2017 to improve access to diagnosis, treatment, and health insurance coverage for patients with rare diseases. However, research on real-world patient experiences and needs is limited, and large-scale studies do not necessarily reflect the characteristics of specific populations, limiting their utility for informing legislative efforts. Thus, the present study aimed to evaluate the real-world experiences of patients in Pennsylvania with rare diseases, identify barriers to diagnosis and treatment, and assess the efficacy of existing systems to inform targeted legislative and health care initiatives.

## Methods

### Guidelines

The design and reporting of this study was supported by the Strengthening the Reporting of Observational Studies in Epidemiology guidelines.^29^

### Data Source

PARDAC initiated the Rare Disease Needs Assessment Survey to understand the needs and challenges faced by individuals, families, and caregivers affected by rare diseases across Pennsylvania. The Western Institutional Review Board determined this study was exempt from institutional review board review because the research included only interactions involving survey procedures and the information obtained was recorded by the investigator in such a manner that the identity of the human subjects cannot readily be ascertained, directly or through identifiers linked to the subjects. The survey was administered through Survey Monkey and disseminated through social media, including Facebook, X (formerly Twitter), and Instagram (47.1% of respondents); email (11.3% of respondents); health care providers’ offices (6.2% of respondents); rare disease organizations (27.5% of respondents); and other forms of media (7.9%). All participants provided written informed consent for the collection and use of the survey data for research purposes. Sample bilingual advertisements were displayed in health care settings (eFigure 1 in Supplementary Material).

### Survey Participants

Participants included patients with rare diseases, parents or guardians of minors with rare diseases, and advocates for patients with rare diseases. We collected responses from the survey from September 22, 2020, through January 3, 2023.

### Outcomes

The primary outcome of this study was a survey of the scope of challenges faced by patients with rare diseases in obtaining accurate diagnoses and care in Pennsylvania.

### Variables and Survey Design

This study used a cross-sectional survey design with data from a single time point for each respondent. PARDAC designed the survey and beta tested it in conjunction with patients and caregivers, clinicians, public health professionals, researchers, legislators, and members of patient advocacy organizations. The survey contained 47 multiple-choice questions and free-text entries for participants to self-report their rare diseases and share additional information about their experiences. Respondents could choose to skip questions (eFigure 2 in Supplementary Material).

### Data Review and Statistics

Together with the Pennsylvania Health Care Cost Containment Council, we manually reviewed and cleaned the data. We combined the county of residence with data from the Center for Rural Pennsylvania to classify each county as rural or urban. We sourced county median annual household income (2019-2023) from the National Institutes of Health HD*Pulse* data portal.^30^ To classify the self-reported diagnoses, we used ChatGPT (GPT-3.5, accessed January 2024), prompting it to classify the primary diagnosis into 5 broad categories without prespecification: autoimmune disorders, neurologic conditions, chromosomal abnormalities, rare genetic disorders, and other conditions. We verified results through manual inspection of a subset of records, ensuring robust agreement with human classification into the resulting categories. We binarized time to an accurate diagnosis as <1 year or ≥1 year for selected analyses. We excluded fully blank responses but otherwise allowed missing data, and we conducted all analyses by using available responses for variables of interest. Given the reported challenges that many respondents faced in receiving a formal diagnosis, the inevitable knowledge gaps among patients, and incomplete epidemiological data for some conditions to determine their status as a rare disease, we chose not to screen responses based on patient-reported disease names. We conducted subsequent statistical analyses in R version 4.4.2 (R Foundation for Statistical Computing) and Stata version 17.0 (StataCorp LLC). We used the χ^2^ test and considered *P* < .05 to be significant.

## Results

A total of 1214 people responded to the survey, including patients with rare diseases, family members, and advocates. Most respondents were adults aged 21 to 64 years (61.2%), identified only as White (90.8%) and female (67.7%) (Table 1). Health insurance coverage was split between private forms of commercial health insurance (63.1%) and government-funded sources, including Medicare and Medicaid (61.3%). Respondents were from 62 of the 67 counties in Pennsylvania (Figure), and most were from urban counties (73.3%).

**Table 1.** Characteristics of respondents (N = 1214) and diagnostic challenges in the PARDAC Rare Disease Needs Assessment Survey, Pennsylvania, September 2020 through January 2023

Characteristic	Total no. of responses^a^	No. (%)
Gender identity		
Male	1197	371 (31.0)
Female		819 (68.4)
Nonbinary		7 (0.6)
Age, y		
<1	1211	8 (0.7)
1-5		74 (6.1)
6-10		82 (6.8)
11-20		143 (11.8)
21-31		116 (9.6)
31-40		169 (14.0)
41-50		165 (13.6)
51-60		189 (15.6)
61-64		102 (8.4)
≥65		163 (13.5)
Race and ethnicity		
American Indian/Alaska Native	—^b^	11 (0.9)
Asian		23 (1.9)
Black or African American		16 (1.3)
Hispanic or Latino		19 (1.6)
Middle Eastern or North African		6 (0.5)
Native Hawaiian/Pacific Islander		2 (0.2)
White		1140 (94.6)
Other		18 (1.5)
Health insurance type		
Through employer	—^b^	684 (56.8)
Self-purchased		78 (6.5)
Medicare		342 (28.4)
Medicaid/other government program		398 (33.0)
None		8 (0.7)
Other		64 (5.3)
Annual spending related to rare disease care, $		
<5000	842	497 (59.0)
5000-10 000		243 (28.9)
10 001-25 000		75 (8.9)
25 001-50 000		16 (1.9)
50 001-75 000		7 (0.8)
75 001-100 000		0
>100 000		4 (0.5)
Does the patient have a specific diagnosis?		
Yes: 1 diagnosis	1213	958 (79.0)
Yes: >1 diagnosis		193 (15.9)
No		62 (5.1)
Relationship to patient		
Patient	1210	755 (62.4)
Parent		348 (28.8)
Spouse		40 (3.3)
Other family member		44 (3.6)
Legal guardian		8 (0.7)
Other advocate		15 (1.2)
No. of health care providers seen from first signs/symptoms to final diagnosis		
1 or 2	1067	235 (22.0)
3 or 4		360 (33.7)
5 or 6		173 (16.2)
7 or 8		92 (8.6)
9 or 10		37 (3.5)
>10		170 (15.9)
No. of inaccurate diagnoses		
0	1065	296 (27.8)
1 or 2		376 (35.3)
3 or 4		226 (21.2)
5 or 6		66 (6.2)
>6		101 (9.5)
Time from first symptoms to accurate diagnosis		
<3 mo	1061	217 (20.5)
3-6 mo		119 (11.2)
6-12 mo		112 (10.6)
1-2 y		133 (12.5)
2-3 y		100 (9.4)
4-5 y		66 (6.2)
>5 y		314 (29.6)
Distance needed to travel for medical care in Pennsylvania, miles		
<25	1011	337 (33.3)
25-50		257 (25.4)
50-100		151 (14.9)
>100		155 (15.3)
Need to travel outside Pennsylvania		111 (11.0)
County type		
Rural	1182	316 (26.7)
Urban		866 (73.3)
County median annual household income, $^c^		
<60 000	1182	46 (3.9)
60 000-70 000		241 (20.4)
70 001-80 000		369 (31.2)
>80 000		526 (44.5)

Abbreviation: PARDAC, PA Rare Disease Advisory Council.

a The total number of respondents is listed for each characteristic of mutually exclusive categories.

b Respondents were permitted to select all that applied.

c Respondents were asked to indicate county of residence. County median annual household income (2019-2023) was sourced from the National Institutes of Health HD*Pulse* data portal.^30^

**Figure.**
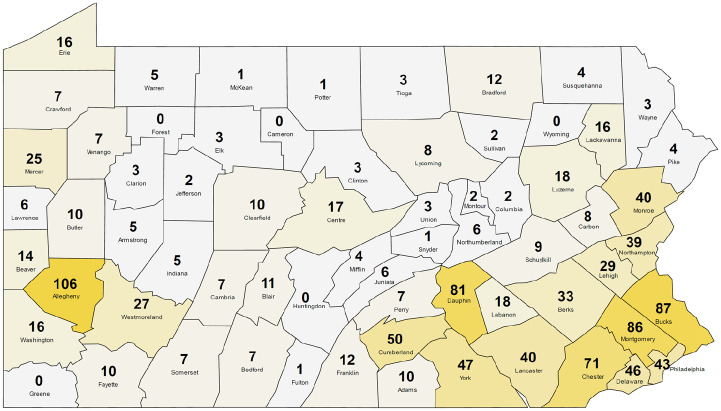
Number of PA Rare Disease Advisory Council respondents to the Rare Disease Needs Assessment Survey from each county, Pennsylvania, September 2020–January 2023. Counties are shaded by relative number of respondents for visualization purposes.

Most respondents indicated only 1 primary diagnosis (74.5%), although some respondents noted 2 (15.3%) or 3 (10.2%) diagnoses. The range of self-reported conditions was broad and heterogeneous. Some conditions were established genetic syndromes, such as 17q12 deletion syndrome, Ehlers–Danlos syndrome, and Charcot–Marie–Tooth disease. Other conditions were rare forms of neoplasia such as appendix cancer and congenital disorders such as agenesis of the corpus collosum and amniotic band syndrome. Some respondents listed broader conditions that would not necessarily qualify as rare diseases, such as gastroparesis and fibromyalgia, and others listed inflammatory or infectious diseases, such as Lyme disease/Babesiosis/Bartonella/Ehrlichia. Among the most frequently reported diseases were immune disorders including common variable immunodeficiency. Approximately 19% of respondents had an autoimmune disorder, 14% had a neurologic condition, 15% had a chromosomal alteration, and 18% had a genetic disorder (Table 2). One-third of respondents (33.5%) indicated that their conditions were genetic or inherited.

**Table 2.** Characteristics and personal impact of respondents to the PARDAC Rare Disease Needs Assessment Survey, by time to accurate diagnosis, Pennsylvania, September 2020 through January 2023^a^

Characteristic	<1 y to diagnosis	≥1 y to diagnosis	*P* value^b^
Sex			.11
Male	146 (33.0)	170 (28.2)	
Female	296 (67.0)	433 (71.8)	
Age, y			<.001
0-20	143 (31.9)	124 (20.3)	
20-50	159 (35.5)	239 (39.1)	
>50	146 (32.6)	249 (40.7)	
Race and ethnicity^c^			
American Indian/Alaska Native	4 (0.9)	6 (1.0)	.99
Asian	8 (1.8)	12 (2.0)	.99
Black or African American	4 (0.9)	10 (1.6)	.44
Hispanic or Latino	5 (1.1)	9 (1.5)	.82
Middle Eastern or North African	1 (0.2)	4 (0.7)	.58
Native Hawaiian/Pacific Islander	0	1 (0.2)	.99
White	430 (96.0)	574 (93.6)	.12
Other	3 (0.7)	14 (2.3)	.07
County type			.75
Rural	125 (28.2)	162 (27.1)	
Urban	318 (71.8)	435 (72.9)	
Disease category			.77
Autoimmune disorder	84 (19.1)	113 (19.0)	
Neurologic condition	66 (15.0)	97 (16.2)	
Chromosomal alteration	57 (13.0)	91 (15.2)	
Genetic disorder	155 (35.2)	194 (32.5)	
Other	78 (17.7)	102 (17.1)	
No. of health care providers seen from first signs/symptoms to final diagnosis			<.001
1 or 2	184 (41.1)	48 (7.8)	
3 or 4	173 (38.6)	185 (30.2)	
≥5	91 (20.3)	379 (61.9)	
No. of incorrect diagnoses			<.001
0	210 (47.1)	81 (13.3)	
1 or 2	185 (41.5)	191 (31.3)	
≥3	51 (11.4)	339 (55.5)	
Personal costs incurred by patient or family related to rare disease care			.004
Yes	300 (76.9)	468 (84.6)	
No	90 (23.1)	85 (15.4)	
Annual spending related to care of rare disease, $			<.001
<5000	216 (67.7)	255 (53.5)	
5000-10 000	75 (23.5)	156 (32.7)	
>10 000	28 (8.8)	66 (13.8)	
Needed to leave a job or reduce work hours			<.001
Yes	223 (56.6)	381 (68.3)	
No	171 (43.4)	177 (31.7)	
Days off from work or school needed for medical reasons, per month			.004
0-2	248 (64.6)	299 (56.0)	
3-5	77 (20.1)	106 (19.9)	
≥6	59 (15.4)	129 (24.2)	
Need to travel out of state for medical care			.01
Yes	99 (25.0)	183 (32.7)	
No	297 (75.0)	377 (67.3)	

Abbreviation: PARDAC, PA Rare Disease Advisory Council.

a Time to diagnosis was binarized based on survey results. The maximum number of respondents was 1214 for each category.

b *P* values were computed using the χ^2^ test, with *P* < .05 indicating significance.

c Respondents could check multiple races and ethnicities. As such, *P* values were computed separately for each race comparing respondents who selected each race with those who did not select that race.

Nearly half (45.2%) of respondents indicated they did not receive a rare disease diagnosis for ≥2 years after their first symptoms appeared; 29.6% of respondents indicated that it took >5 years to receive a diagnosis (Table 1). Similarly, 44.2% of participants indicated that they visited ≥5 health care providers before receiving their final diagnosis; 15.9% indicated that they saw >10 health care providers before their final diagnosis. Consequently, 36.9% reported ≥3 incorrect diagnoses and 15.7% reported ≥5 incorrect diagnoses before the final correct diagnosis. Notably, the diagnostic time (across all time categories) was not significantly different between category of rare disease (*P* = .55), urban and rural counties (*P* = .64), or county median annual household income (*P* = .41). Diagnostic time was significantly associated with sex (*P* = .04), with males more likely than females to have an accurate diagnosis in <3 months (24.0% vs 18.8%) and females more likely than males to take ≥5 years to obtain a diagnosis (32.4% vs 22.8%). When stratifying diagnosis time as <1 year or ≥1 year (Table 2), we found that older patients were more likely than younger patients to have longer diagnosis times and that a longer diagnosis time was associated with a larger number of health care providers seen and previous incorrect diagnoses, as expected (*P* < .001). No race or ethnicity was associated with diagnosis time.

Nearly half (43.5%) of respondents indicated transportation challenges in getting to appointments, work, or school in their communities. In addition, 30.2% traveled more than 50 miles for health care, 29.5% needed to travel out of state for any medical care, and 11.0% were unable to access care for their rare diseases in the Commonwealth of Pennsylvania. The need to travel out of state was significantly associated with long diagnosis times (*P* = .01).

Approximately half of respondents (48.7%) lacked the counseling support they needed, and 72.9% indicated that they were uninformed about patient organizations or support groups at the time of diagnosis (Table 3). At the time of diagnosis, 37.0% of respondents indicated that they did not receive the right amount of information about their disease, and 20.5% said they did not understand the information provided by their health care providers. Most (78.8%) respondents indicated that they currently had access to needed information about their rare disease. One-third (30.2%) of patients said they did not receive timely access to specialists and clinics.

**Table 3.** Respondent perspectives on efficacy of care based on time to accurate diagnosis through the PARDAC Rare Disease Needs Assessment Survey, Pennsylvania, September 2020 through January 2023^a^

Time to diagnosis	Agree	Somewhat agree	Somewhat disagree	Disagree	*P* value^b^
I received the right amount of information about my rare disease at the time of diagnosis.					
Total	289 (27.4)	375 (35.6)	176 (16.7)	214 (20.3)	—
<1 y	138 (31.1)	177 (39.9)	65 (14.6)	64 (14.4)	<.001
≥1 y	147 (24.5)	196 (32.6)	110 (18.3)	148 (24.6)	
I understood the information provided to me by my health care provider about my rare disease diagnosis.					
Total	467 (44.4)	369 (35.1)	112 (10.6)	104 (9.9)	—
<1 y	214 (48.4)	153 (34.6)	40 (9.0)	35 (7.9)	.05
≥1 y	248 (41.3)	213 (35.5)	71 (11.8)	68 (11.3)	
At the time of my diagnosis, I was given information about a patient organization or support group.					
Total	286 (27.1)	—	—	769 (72.9)	—
<1 y	124 (28.1)	—	—	318 (71.9)	.48
≥1 y	156 (25.9)	—	—	446 (74.1)	
I currently have access to needed information about my rare disease.					
Total	834 (78.8)	—	—	224 (21.2)	—
<1 y	372 (83.6)	—	—	73 (16.4)	.001
≥1 y	453 (75.2)	—	—	149 (24.8)	
I received timely testing and treatment after my diagnosis.					
Total	441 (43.8)	292 (29.0)	110 (10.9)	164 (16.3)	—
<1 y	244 (58.0)	115 (27.3)	26 (6.2)	36 (8.6)	<.001
≥1 y	194 (33.7)	172 (29.9)	83 (14.4)	127 (22.0)	
After my diagnosis, I received timely access to specialists and clinics.					
Total	445 (43.6)	267 (26.2)	114 (11.2)	194 (19.0)	—
<1 y	250 (58.3)	98 (22.8)	44 (10.3)	37 (8.6)	<.001
≥1 y	190 (32.8)	167 (28.8)	69 (11.9)	154 (26.6)	
After my diagnosis, I received timely assistance coordinating with specialists and clinics providing care.					
Total	391 (38.7)	270 (26.7)	117 (11.6)	233 (23.0)	—
<1 y	226 (53.6)	104 (24.6)	40 (9.5)	52 (12.3)	<.001
≥1 y	161 (27.9)	162 (28.0)	75 (13.0)	180 (31.1)	
After my diagnosis, I received timely access to medications through health insurance or a drug plan.					
Total	406 (44.3)	243 (26.5)	94 (10.3)	174 (19.0)	—
<1 y	207 (54.6)	95 (25.1)	31 (8.2)	46 (12.1)	<.001
≥1 y	197 (37.2)	147 (27.8)	60 (11.3)	125 (23.6)	
I am aware of approved medications for my specific rare disease or diseases.					
Total	605 (74.1)	—	—	212 (25.9)	—
<1 y	269 (78.7)	—	—	73 (21.3)	.02
≥1 y	331 (70.9)	—	—	136 (29.1)	
I have access to approved medications for my rare disease through my health insurance or drug plan.					
Total	594 (76.3)	—	—	184 (23.7)	—
<1 y	263 (82.2)	—	—	57 (17.8)	.002
≥1 y	325 (72.5)	—	—	123 (27.5)	
It has been difficult or stressful to access medications for my rare disease.					
Total	276 (32.8)	286 (34.0)	95 (11.3)	185 (22.0)	—
<1 y	83 (25.7)	97 (30.0)	49 (15.2)	94 (29.1)	<.001
≥1 y	173 (36.7)	169 (35.9)	45 (9.6)	84 (17.8)	
I have been unable to access medications for my rare disease because of copay costs or lack of coverage.					
Total	259 (35.1)	—	—	478 (64.9)	—
<1 y	76 (26.5)	—	—	211 (73.5)	<.001
≥1 y	165 (39.8)	—	—	250 (60.2)	
I have access to off-label medications through health insurance or drug plan.					
Total	285 (50.7)	—	—	277 (49.3)	—
<1 y	110 (54.5)	—	—	92 (45.5)	.24
≥1 y	158 (48.8)	—	—	166 (51.2)	
I feel that I have the counseling support that I need.					
Total	203 (21.5)	282 (29.8)	178 (18.8)	283 (29.9)	—
<1 y	91 (25.1)	115 (31.7)	67 (18.5)	90 (24.8)	.04
≥1 y	106 (20.0)	151 (28.5)	97 (18.3)	175 (33.1)	

Abbreviation: PARDAC, PA Rare Disease Advisory Council.

a Questions are listed as presented to survey respondents. Time to diagnosis was binarized based on survey results. The maximum number of respondents was 1214 per category.

b *P* values were computed using the χ^2^ test, with *P* < .05 indicating significance.

Most respondents (81.4%) indicated that they or their families had incurred personal costs related to caring for their rare disease (Table 2). About half of respondents (48.5%) reported >$5000 in annual spending related to care for their rare disease; 10.6% reported >$10 000 in annual spending. Annual spending did not vary significantly by race (*P* = .90) or age (*P* = .08); it did vary significantly by sex, with males more likely than females to be in higher spending categories (*P* = .03). It also varied significantly by health insurance type (*P* < .001), with 65.2% of Medicaid users paying $0 to $2000 versus 56.8% of Medicare users or 54.8% of patients with employer-funded health insurance. Furthermore, 63.5% indicated that they needed to leave a job or reduce work hours because of their rare disease or as a family member or caregiver, and 46.2% took unpaid leave for medical reasons. Notably, longer diagnosis times were associated with increased annual spending and challenges affecting the ability to work (Table 2).

One-quarter of respondents (24.9%) said that they were unable to access medications because of copay costs or lack of coverage, and 46.3% were not aware of programs to help cover medication costs. More than 30% of respondents reported having long-term disability related to their rare disease, and 73.9% of respondents who submitted a long-term disability benefit application had difficulties during the approval process. Applications for social security disability benefits were the most common request submitted (89.4%). Lastly, longer diagnosis times were associated with poorer patient experiences across a range of survey questions (Table 3). These experiences included patient education and access to testing, treatment, and specialists. The only responses that were not significantly different based on diagnosis time were whether the patient understood the information presented and whether the patient was given access to information about patient organizations or support groups (Table 3).

## Discussion

In this study, we used a community-based cross-sectional survey to explore the experiences of individuals in Pennsylvania who have been diagnosed with a rare disease. We found that these experiences varied widely—some individuals received a clear diagnosis and expedient treatment, while others went through years of misdiagnoses. With more than 1200 respondents representing more than 660 rare diseases, the Rare Disease Patient Needs Assessment Survey sheds light on the diagnostic odyssey, treatment journey, and the profound effect on quality of life for patients and their families. The findings underscore the urgent need for increased awareness, support, and advocacy for individuals affected by rare diseases. Although this survey was conducted in Pennsylvania to inform state-level policy development, the experiences reported align with national and international findings on the diagnostic odyssey, suggesting that these barriers and unmet needs are widespread and systemic.^31-34^

Previous large-scale surveys have similarly reported prolonged diagnostic timelines, frequent misdiagnoses, and challenges in accessing appropriate care for patients with a rare disease.^16,35-37^ For example, the landmark EURODIS survey of 12 000 patients in Europe found that 25% of people waited 5 to 30 years for an accurate diagnosis, and 40% received at least 1 incorrect diagnosis.^34^ Likewise, the Rare Disease Impact Report identified that patients often see multiple health care providers and undergo extensive testing before reaching a diagnosis, with substantial effects on quality of life and financial burden.^31^ Our study adds to the literature by revealing that delays in diagnosis were negatively associated with a range of patient perceptions and quality-of-life metrics across a wide breadth of questions, offering empirical evidence of associations that are often described anecdotally or qualitatively in prior work.^17,38-40^ These associations include challenges with employment, increased travel demands, and difficulties in coordinating with specialists and receiving access to necessary testing and treatment. Previous studies have similarly noted that such delays contribute not only to emotional distress but also to long-term logistical and financial hardship for families.^41-44^ While this observation is correlative, increased uncertainty and frustration along with multiple incorrect diagnoses and specialist visits may complicate a patient’s medical history, creating challenges for coordination of care even after a diagnosis has been reached. Furthermore, we observed that pediatric patients had markedly reduced diagnosis time compared with adult patients, in contrast with the large Rare Barometer European survey.^45^ Genetic diseases that can be definitively diagnosed by karyotyping or sequencing often present early in life, although increased vigilance and social support for younger patients may help to avoid longer diagnostic delays, contributing to this observation.^37,46,47^ Collectively, our findings reinforce the need for improved early diagnosis and care coordination to reduce the financial and emotional burden on patients and families.

Other reports have revealed differences in perception between patients and physicians. For example, patients with rare diseases faced substantially more difficulties in receiving accurate diagnoses compared with the physicians’ expectations.^41^ Physicians may underestimate the complexity of the disease process, leading to underuse of appropriate diagnostics or delayed referrals to specialists, which can take patients months to access.^48,49^ Previous reports have described the importance of rapid referral,^50,51^ suggesting that it would benefit patients to begin the referral process early in the disease course while other diagnostic and treatment approaches are attempted. A substantial portion of rare diseases remain undiagnosed or misdiagnosed because of the ambiguous and multifactorial nature of the genetic and molecular mechanisms involved.^52^ Even then, for some patients, receiving a diagnosis signals the beginning of an even longer therapeutic journey, which can take years or decades if therapies have not yet been developed. The postdiagnostic phase for individuals with rare diseases often includes limited or no available treatments, off-label prescribing, and prolonged efforts to access clinical trials or compassionate use programs.^43,53^ Consequently, while diagnosis is a critical milestone, it frequently marks a transition in a new phase of uncertainty and navigation through complex treatment landscapes.

Taken together, this study highlights an effective model for how state and local governments can gather critical, patient-centered data to inform policy and resource allocation for people living with rare diseases. Several actionable recommendations emerged from our findings:

1. Expand access to genetic testing by establishing state-funded rare disease diagnostic programs, which would help reduce diagnostic delays, particularly in medically underserved and rural communities.
2. Enhance health care provider education by incorporating rare disease recognition, referral networks, and diagnostic best practices into Continuing Medical Education opportunities, addressing knowledge gaps among frontline health care providers. PARDAC is currently developing a Pennsylvania-specific resource center to catalog available resources and specialists in the state.
3. Invest in transportation assistance programs, which would improve access to timely care for patients facing geographic or financial barriers.

When PARDAC was formed in 2017, Pennsylvania was 1 of 5 states in the United States with a rare disease advisory council. As of 2025, Pennsylvania was 1 of 30 states with a rare disease advisory council, and it works closely with the National Organization of Rare Disorders. Given the intrinsic knowledge gap between patients and health care providers, future surveys would benefit from the inclusion of primary care and specialist physicians to better understand the discrepancies between physicians’ expectations and the patient experience. Moreover, linking patient-facing surveys to electronic health records would facilitate precise documentation of the rare disease, abrogating errors in patient recall and facilitating a more detailed analysis of issues relevant to specific categories of rare diseases.

### Limitations

This study had several limitations. First, although the survey reached a diverse population of patients with rare diseases spanning multiple demographic characteristics, it was primarily distributed through health care providers and social media, which may have biased the respondent pool toward individuals already connected to the medical system, such as White and urban populations. Subsequent studies are needed to enhance the visibility of underrepresented racial and ethnic groups and rural communities in the rare disease landscape to reduce health care disparities. Second, potential response errors in self-reported annual spending and health insurance coverage as well as skipped questions could have led to measurement bias. In addition, ongoing surveys are exploring other variables such as disease severity, access to health care facilities, socioeconomic status, and comorbidities to provide a comprehensive understanding of the factors influencing annual spending among these patients and control for potentially confounding effects. Third, the survey data and corresponding analysis were intrinsically correlative, limiting the ability to draw causal relationships between key variables, such as annual spending and health insurance type. Similarly, further research is needed to identify how changes in health care policy, health insurance coverage, or rare disease treatments over time could influence spending patterns.

## Conclusion

This study highlights key challenges faced by people with rare diseases in Pennsylvania, including delayed diagnosis, limited access to specialists, financial burdens, and lack of awareness about support resources. Addressing these issues through improved diagnostics, expanded access to care, and targeted policy changes is essential to enhancing patient outcomes and quality of life. The findings from this community-based study serve as a model for other states and localities to identify and address similar gaps in rare disease care.

## Supplemental Material

sj-pdf-1-phr-10.1177_00333549251362711 – Supplemental material for The Experiences of Patients With Rare Diseases in Pennsylvania: A Community-Based Study Supplemental material, sj-pdf-1-phr-10.1177_00333549251362711 for The Experiences of Patients With Rare Diseases in Pennsylvania: A Community-Based Study by Jonathan H. Sussman, Mert Marcel Dagli, Shira L. Wald, Saad S. Akhtar, Keanu Natan, Jessica A. Xu, Alexander Li, Brian Dawson and William C. Welch in Public Health Reports®
