# Supplementary material for: The Experiences of Patients With Rare Diseases in Pennsylvania: A Community-Based Study
Source: Public Health Rep. 2025 Sep 7;140(5-6):605–16. doi: 10.1177/00333549251362711 (PMC12414988; doi:10.1177/00333549251362711)
Supplement: Supplementary file 1 [file sj-pdf-1-phr-10.1177_00333549251362711.pdf]

Supplementary Materials

Survey Questions

Q1. ELECTRONIC CONSENT: Clicking on the "Agree" button indicates that: You have read the above information; You voluntarily agree to participate; You are 18 years of age or older; You reside in Pennsylvania.

Response Choices
Agree
Disagree

Q2. Who is completing this questionnaire? (If the person with the rare disease is under the age of 18, a parent or legal guardian can complete this survey on behalf of the person with the rare disease. A family member or other advocate may also complete this survey on behalf of the person with rare disease over age 18.)

Response Choices
Person with rare disease
Parent of person with rare disease
Spouse of person with rare disease
Other family member (not parent or spouse) of person with rare disease
Legal guardian of person with rare disease
Other advocate of person with rare disease

Q3. How did you hear about this survey? (check all that apply)

Response Choices
Pennsylvania Rare Disease Advisory Council
NORD (National Organization of Rare Diseases)
Another rare disease organization
Health care provider's office
Media coverage
Email
Social media (such as Facebook or Twitter)
Other (please specify)

Q4. What is the gender identification of the person with rare disease?

Response Choices
Male
Female
Non-binary
Prefer not to answer

Q5. Does the person with rare disease identify as transgender?

Response Choices
Yes
No

Q6. What is the age of the person with rare disease?

Response Choices
Birth to 12 months
1 to 5 years
6 to 10 years
11 to 20 years
21 to 30 years
31 to 40 years
41 to 50 years
51 to 60 years
61 to 64 years
65 and over

Q7. What is the race/ethnicity of the person with rare disease? (check all that apply)

Response Choices
American Indian or Alaska Native
Asian
Black or African American
Hispanic or Latino
Middle Eastern or North African
Native Hawaiian or other Pacific Islander
White
Some other race, ethnicity or origin

Q8. In what county does the person with rare disease reside?

Response Choices - Select from one of 67 PA Counties

Q9. What type of health insurance does the person with the rare disease have? (check all that apply)

Response Choices
Insurance through employer (self or family member)
Self-purchased insurance
Medicare
Medicaid or other government program
No insurance
Other (please specify)

Q10. Is your rare disease a genetic (or inherited) condition?

Response Choices
Yes
No
There may be a genetic component
Unknown

Q11. Do you have a specific diagnosis for your rare disease?

Response Choices
Yes, I have a rare disease diagnosis
Yes, I have more than one rare disease diagnosis
No, I do Not yet have a specific rare disease diagnosis

Q12. Please share the name of your rare disease or diseases. You may leave this answer blank if you prefer not to answer.

Response Choices
My rare disease is:
I have a second rare disease which is:
I have a third rare disease which is:

Q13. How long after the first symptoms or suspicion of disease did it take to get an accurate diagnosis?

Response Choices
less than 3 months
3 to 6 months
6 to 12 months
1 to 2 years
2 to 3 years
4 to 5 years
5 years or more

Q14. How many healthcare providers did you see between the first signs or symptoms and the final diagnosis?

Response Choices
1-2
3-4
5-6
7-8
9-10
More than 10

Q15. How many incorrect or inaccurate diagnoses were made before the final correct diagnosis?

Response Choices
0
1-2
3-4
5-6
More than 6

Q16. I received the right amount of information about my rare disease at the time of diagnosis.

Response Choices
Agree
Somewhat Agree
Somewhat Disagree
Disagree

Q17. I understood the information provided to me by my healthcare provider about my rare disease diagnosis.

Response Choices
Agree
Somewhat Agree
Somewhat Disagree
Disagree

Q18. At the time of my diagnosis, I was given information about a patient organization or support group.

Response Choices
Agree
Disagree

Q19. I currently have access to needed information about my rare disease.

Response Choices
Agree
Disagree

Q20. I received timely testing and treatment after my diagnosis.

Response Choices
Agree
Somewhat Agree
Somewhat Disagree
Disagree
Not applicable

Q21. After my diagnosis, I received timely access to specialists and clinics.

Response Choices
Agree
Somewhat Agree
Somewhat Disagree
Disagree
Not applicable

Q22. After my diagnosis, I received timely assistance coordinating with specialists and clinics providing care.

Response Choices
Agree
Somewhat Agree
Somewhat Disagree
Disagree
Not applicable

Q23. After my diagnosis, I received timely access to medications through health insurance or a drug plan.

Response Choices
Agree
Somewhat Agree
Somewhat Disagree
Disagree
Not applicable

Q24. I am aware of approved medications for my specific rare disease or diseases.

Response Choices
Agree
Disagree

Q25. I have access to approved medications for my rare disease through my health insurance or drug plan.

Response Choices
Agree
Disagree
Not applicable

Q26. I received timely assessment and testing in search of a specific diagnosis.

Response Choices
Agree
Somewhat Agree
Somewhat Disagree
Disagree
Not applicable

Q27. I received timely access to specialists and clinics through health insurance.

Response Choices
Agree
Somewhat Agree
Somewhat Disagree
Disagree
Not applicable

Q28. I received timely assistance coordinating with specialists and clinics providing care.

Response Choices
Agree
Somewhat Agree
Somewhat Disagree
Disagree
Not applicable

Q29. I received timely access to medications through my health insurance or drug plan.

Response Choices
Agree
Somewhat Agree
Somewhat Disagree
Disagree
Not applicable

Q30. I received timely access to the following non-drug treatments through health insurance when needed:

Response Choices - Select Agree - Disagree - N/A - for choices below
Medical Foods
Physical Therapy
Occupational Therapy
Procedures Or Surgery
Supplemental Nursing Care (Private-Duty Or Shift-Care Nursing)
Palliative Care

Q31. It has been difficult or stressful to access medications for my rare disease.

Response Choices
Agree
Somewhat Agree
Somewhat Disagree
Disagree
Not applicable

Q32. I have been unable to access medications for my rare disease because of copay costs or lack of coverage.

Response Choices
Agree
Disagree

Q33. I have access to off-label medications through health insurance or drug plan. (off-label means that the medication is being used in a manner not specified in the FDA's approved package label or insert for the medication. This might include use for a diagnosis not listed as approved, or use in an unapproved age group, dosage or route of administration)

Response Choices
Agree
Disagree
Not applicable

Q34. There are programs that help patients and their families with medication costs. Which of these are you aware of? (check all that apply)

Response Choices
Copay / Coinsurance Assistance Provided By Manufacturers
Patient Assistance Programs Through Manufacturers
Assistance Through Third Parties Such As Foundations
None Of The Above

Q35. Which of these medication assistance programs have you used? (check all that apply)

Response Choices
Copay / Coinsurance Assistance Provided By Manufacturers
Patient Assistance Programs Through Manufacturers
Assistance Through Third Parties Such As Foundations
None Of The Above

Q36. How far do you need to travel within Pennsylvania for medical care related to your rare disease? (if more than one clinic or institution, refer to the longest distance)

Response Choices
less than 25 miles
25 – 50 miles
50 – 100 miles
more than 100 miles
I am unable to access care for my rare disease within the state of Pennsylvania

Q37. Do you need to travel out-of-state for any of your medical care related to your rare disease?

Response Choices
Yes
No

Q38. Has transportation for medical appointments, work or school been challenging for you? (for the rare disease patient, family or caregivers)

Response Choices
Yes
No

Q39. How often are days off work or school needed for medical reasons (per month, on average)? (for the rare disease patient, family or caregivers)

Response Choices
0-2 days per month
3-5 days per month
6-10 days per month
>10 days per month

Q40. Do you often need to take unpaid leave for medical reasons? (for rare disease patient, family or caregiver)

Response Choices
Yes
No
Not applicable

Q41. Have you ever had to leave a job or reduce work hours (such as going from full time to part-time) as a result of your rare disease? (for rare disease patient, family or caregiver)

Response Choices
Yes
No

Q42. Do you feel that you have the counseling support that you need?

Response Choices
Agree
Somewhat Agree
Somewhat Disagree
Disagree
Not applicable

Q43. Have you had difficulties with emergency medical care (emergency department care or ambulance/paramedic services) for your rare disease?

Response Choices
Yes
No
Not applicable

Q44. Have you had difficulties with inpatient care for your rare disease?

Response Choices
Yes
No
Not applicable
Please comment if desired.

Q45. If you have a long-term disability related to your rare disease, did you have difficulties with approval of your long-term disability benefit application?

Response Choices
Yes, but eventually approved
Yes, still not approved
No, the process was smooth
Not applicable
Please comment if desired.

Q46. If you have applied (approved or not) for long-term disability benefits, check all that apply:

Response Choices
Private Disability Insurance
Employer Disability Insurance
Social Security Disability

Q47. Do you and your family incur personal costs related to care for your rare disease?

Response Choices
Yes
No

Q48. What is the amount that your family spends annually related to care for your rare disease?

Response Choices

< $5,000 per year

$10,001- $25,000 per year

$25,001 - $50,000 per year

$50,001 - $75,000 per year

$75,001- $100,000 per year

Over $100,000

Q49. If desired, please share any other comments about your needs or experiences.

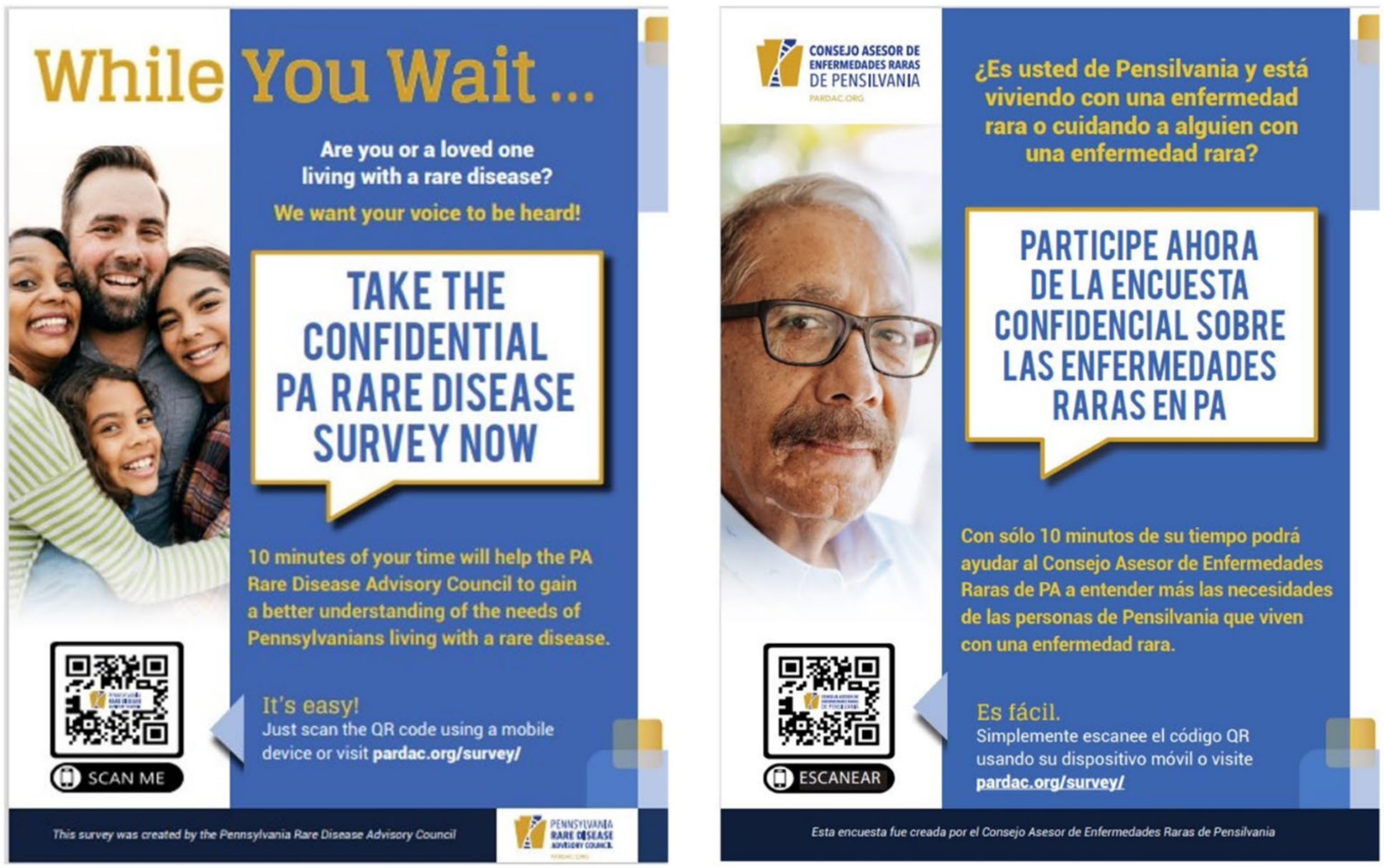

Supplementary Figure 1. Examples of bilingual (English/Spanish) advertising campaign displayed in healthcare offices.
